# Multiple cycles of intermittent chemotherapy in metastatic androgen-independent prostate cancer

**DOI:** 10.1038/sj.bjc.6602198

**Published:** 2004-10-05

**Authors:** T M Beer, M Garzotto, W D Henner, K M Eilers, E M Wersinger

**Affiliations:** 1 Department of Medicine, Division of Hematology and Medical Oncology, Oregon Health & Science University, Mail Code CR145, 3181 SW Sam Jackson Park Road, Portland, OR 97239, USA; 2 Division of Urology, Oregon Health & Science University and Portland VA Medical Center, 3710 SW US Veterans Hospital Road, Mail Code: P3GU Portland, OR 97207, USA

**Keywords:** docetaxel, calcitriol, vitamin D, prostate cancer, chemotherapy

## Abstract

Recently, completed phase III studies demonstrated a survival benefit for a fixed number of cycles of docetaxel-containing chemotherapy treatment of androgen-independent prostate cancer (AIPC). Management of patients who respond well to initial chemotherapy for AIPC remains ill-defined. We previously reported that in a select group of such patients, retreatment with the same regimen was feasible and was associated with quality of life gains. Here, we report that multiple cycles of such intermittent chemotherapy are feasible. We prospectively tested intermittent chemotherapy in eight AIPC patients responding to calcitriol plus docetaxel who reached a serum prostate-specific antigen (PSA) <4 ng ml^−1^ (22% of the 37 patients who were initially treated with this regimen). Chemotherapy was suspended until a rise in PSA ⩾50% and 1 ng ml^−1^. The median duration of the first treatment holiday was 20 weeks (13–74 weeks) and all patients retained sensitivity to retreatment. Four patients were eligible for a second chemotherapy holiday, and the median duration was 21 weeks (17–28 weeks). Two patients elected to take a third chemotherapy holiday, which lasted 10 and 28 weeks. The median time to treatment failure was 26.5 months (95% CI 23.6–29.4 months), and the median survival is 41 months (95% CI 33.7–48.3 months). Multiple cycles of intermittent chemotherapy interrupted by clinically meaningful treatment holidays are feasible in a subset of AIPC patients treated with this docetaxel-containing regimen. Intermittent chemotherapy for AIPC is feasible and deserves further study.

The optimal duration of chemotherapy for androgen-independent prostate cancer (AIPC) remains uncertain. Recently, a survival benefit for treatment with docetaxel-containing chemotherapy was demonstrated in two large phase III clinical trials (Eisenberger *et al*, 2004; Petrylak *et al*, 2004). Patients were treated for a fixed number of cycles and their total treatment lasted 30 and 36 weeks in these two studies. As these studies are likely to define the standard of care in metastatic AIPC, it is likely that patients who receive a recommendation for chemotherapy will be treated for a fixed number of cycles and a significant fraction of these patients will be responding to treatment when they complete the initial course of chemotherapy.

Available studies provide no guidance regarding the treatment of these patients after an initial course of therapy is completed. Indeed, relatively little information about retreatment with the same chemotherapy after a robust initial response to treatment in any tumour type is available. In a recent review, Cara and Tannock identified 15 published reports of retreatment after initial complete response in a range of solid and haematologic malignancies. Response rates to retreatment with the same regimen ranged from 18 to 100%. None of these studies included prostate cancer patients (Cara and Tannock, 2001).

We previously reported that a single, clinically meaningful chemotherapy holiday was feasible and was associated with quality of life gains in a select group of patients who responded well to initial docetaxel-containing chemotherapy (Beer *et al*, 2003b). We now provide longer-term outcome information about these patients and report our experience with multiple cycles of chemotherapy interrupted by treatment holidays.

In the context of a phase II clinical trial of high-dose calcitriol and docetaxel in AIPC, we developed and prospectively evaluated an intermittent chemotherapy protocol. The rationale for calcitriol plus docetaxel has been previously described (Beer *et al*, 2003a) and outcomes with the first chemotherapy holiday were previously reported (Beer *et al*, 2003b). This report is focused on long-term outcome in patients treated with intermittent chemotherapy.

## MATERIALS AND METHODS

Detailed eligibility criteria and treatment regimen have been previously reported (Beer *et al*, 2003a). Men with chemotherapy-naïve, metastatic AIPC were treated with calcitriol (Rocaltrol® 0.5 *μ*g capsules, Roche Pharmaceuticals, Nutley, NJ) 0.5 *μ*g kg^−1^ on day 1, followed by docetaxel (Taxotere®, Aventis Pharmaceuticals, Bridgewater, NJ) 36 mg m^−2^ intravenously over 15–30 min on day 2 of each treatment week. Dexamethasone 8 mg orally was given 12 and 1 h prior to and 12 h after docetaxel. Treatment was administered weekly for 6 consecutive weeks on an 8-week cycle.

Patients who met criteria for prostate-specific antigen (PSA) response (50% reduction confirmed 4 weeks apart) and reached a serum PSA<4 ng ml^−1^ were eligible for intermittent chemotherapy. During the chemotherapy holiday, a serum PSA and clinical examination was performed every 4 weeks. Imaging was repeated every 8 weeks in patients with measurable disease. Chemotherapy was resumed after a confirmed PSA increase of 50% and of at least 1 ng ml^−1^ (modified to 2 ng ml^−1^ during the course of the study) or for any other evidence of disease progression.

After chemotherapy was resumed, patients whose response to retreatment allowed them to meet eligibility criteria again could have additional chemotherapy holidays. The number of such holiday and retreatment cycles was not restricted. The study was approved by the IRB of the Oregon Health & Science University and Portland VA Medical Center. Informed consent was obtained from all participants.

## RESULTS

Of 37 patients, 11 met eligibility for intermittent chemotherapy after responding to initial treatment, which lasted for a median of 45 weeks (range 25–53 weeks). Except for having a lower serum PSA at study entry, these 11 patients did not differ from the remaining 26 patients and their characteristics have been previously published (Beer *et al*, 2003b). Nine patients consented to intermittent chemotherapy. One patient was removed from the study for reasons unrelated to prostate cancer or its treatment. Therefore, eight patients were evaluable for outcome with intermittent chemotherapy. Baseline characteristics of these eight patients are reported in Table 1

**Table 1:** Patient characteristics prior to starting chemotherapy

**Characteristic**	**Patients who consented to intermittent therapy**
No. of patients	8
*Age (years)*	
Median (range)	74 (68–81)
*ECOG Performance Status*	
0	5
1	2
2	1
PSA (ng ml^−1^)	
Median (range)	15 (6–553)
*Site of metastases*	
Bone only	6
Bone and lymph nodes	1
Lymph nodes only	1
*Prior radiation*	
External beam to primary	4
External beam to metastases	1
Radiopharmaceuticals	0
*No. of prior hormonal treatments*	
Median (range)	2 (1–3)

PSA=prostate-specific antigen.

.

### First chemotherapy holiday

The median duration of the first treatment holiday was 20 weeks (range 13–74 weeks). All patients resumed therapy as a result of a rising PSA. After resumption of therapy, five patients experienced a confirmed 50% reduction in serum PSA from their postholiday measurement, while three had stabilisation of the serum PSA for at least 24 weeks (range 24–56 weeks). Two patients had measurable disease. Both of these entered the break after a partial response (PR) and retained PR status throughout the treatment holiday.

### Second chemotherapy holiday

Only the patients who had a confirmed 50% reduction in serum PSA in response to their second course of chemotherapy met eligibility criteria for a second chemotherapy holiday. One of these five patients died of unrelated causes. Therefore, four patients participated in a second chemotherapy holiday. The median duration of the second holiday was 21 weeks (range 17–28 weeks). Two of these patients experienced a confirmed 50% reduction in serum PSA from their postholiday measurement in response to the third course of chemotherapy. The other two declined a third round of chemotherapy, one because of persistent treatment-related pleural effusions and the other due to patient preference.

### Third chemotherapy holiday

**Figure 1:**
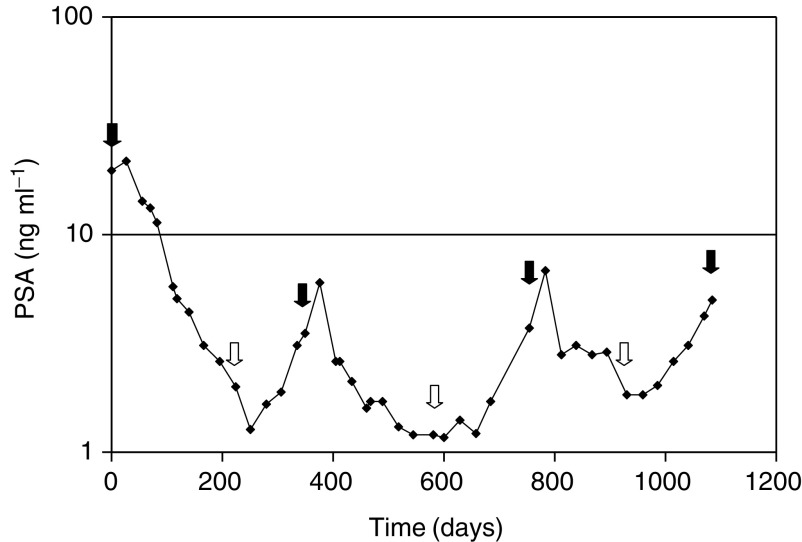
Changes in serum PSA over time in a patient treated with intermittent chemotherapy. Closed arrows indicate when chemotherapy was initiated and open arrows show when chemotherapy was stopped.

The third treatment holiday lasted 10 and 26 weeks. The experience of a patient who has completed three chemotherapy holidays is shown in Figure 1

.

Overall, the median time to treatment failure from the first day of chemotherapy in these eight patients was 26.5 months (95% CI 23.6–29.4 months). Five of the eight patients are alive and the Kaplan–Meier estimate for median survival is 41 months (95% CI 33.7–48.3 months).

## DISCUSSION

While durable complete remission remains an elusive goal in the treatment of AIPC, substantial responses to therapy are frequently reported. The optimal approach to the treatment of AIPC patients with a substantial response to initial chemotherapy remains unclear. We previously reported that retreatment with the same regimen after a single chemotherapy holiday was feasible in such patients, that the chemotherapy holiday was long enough to be clinically meaningful, and that chemotherapy holidays were associated with improvements in common chronic toxicities of chemotherapy.

With longer follow-up, we report here that one half of the patients who chose intermittent chemotherapy were able to continue this treatment approach beyond one cycle. We conclude that in selected patients, multiple courses of docetaxel-based chemotherapy interrupted by chemotherapy holidays are feasible. Additional study is needed to definitively determine the contribution of intermittent chemotherapy to the overall efficacy and toxicity of treatment.
